# Antegrade Kirschner wire fixation for small talar osteochondral fractures: a case report

**DOI:** 10.3389/fsurg.2026.1872423

**Published:** 2026-06-03

**Authors:** Haiqing Wang, Lufeng Yao, Feng Zhang, Lei Huang

**Affiliations:** 1 Department of Foot and Ankle Surgery, Ningbo No. 6 Hospital, Ningbo, Zhejiang, China; 2 Ningbo Clinical Research Center for Orthopedics, Sports Medicine & Rehabilitation, Ningbo, Zhejiang, China

**Keywords:** ankle fracture, case, fixation, osteochondral fracture, talus

## Abstract

Small acute osteochondral fractures (OCFs) of the talus are rare and often undiagnosed. Inaccurate diagnosis or improper management of these injuries can lead to defects in the talar cartilage, increasing the risk of long-term ankle arthritis. The ideal treatment approach for these fractures continues to be a topic of discussion. This report presents a case of antegrade Kirschner wire fixation for an acute small osteochondral fracture on the medial talus, combined with an undisplaced medial malleolus fracture (MMF). The MMF was surgically opened to achieve optimal exposure of the osteochondral fracture. After anatomical reduction of the osteochondral fragment, two 1.0-mm Kirschner wires were inserted antegrade and passed through the opposite skin. They were then removed retrogradely to ensure that the wire tails were flush with the articular surface. Two months postoperatively, radiological imaging confirmed the healing of both the MMF and the osteochondral fragment. The Kirschner wire was then removed percutaneously. At the final follow-up, imaging showed successful osteochondral healing of the talus, with favorable clinical and radiological outcomes.

## Introduction

Osteochondral fractures (OCFs) of the talus are relatively rare, accounting for 0.1% to 1% of all talar fractures (1, 2). However, in the context of ankle fractures, the occurrence of concurrent talar osteochondral injuries is significantly high, ranging from 38% to 79.2% (3–7). Togher et al. (8) reported that 50.9% of all acute ankle fractures are associated with osteochondral lesions of the talus. Severe pain and instability following an ankle fracture can hinder a comprehensive evaluation of secondary injuries. Improper diagnoses or inadequate treatment may result in persistent postoperative pain, talar cysts (9) and an increased risk of long-term ankle arthritis (8). In 1959, Berndt and Harty (10) categorized talar OCFs into four stages: Stage I involves compression of the articular cartilage and subchondral bone; Stage II is characterized by a partially detached osteochondral fragment without displacement; Stage III refers to a completely detached fragment without displacement; and Stage IV involves a completely detached and displaced fragment.

The management of OCFs should be based on a comprehensive assessment of the patient's age, activity level, and the size and location of the osteochondral fragment. Historically, smaller fragments were treated with fragment excision or microfracture techniques (11). However, larger OCFs may require internal fixation, employing methods such as Kirschner wires, absorbable screws, headless screws, or bone pegs (12–16). However, each fixation technique is associated with potential risks, including fixation loosening, fragment displacement, unreliable stabilization, and foreign body reactions. Since approximately 60% of the talar surface is covered with articular cartilage, its blood supply is limited (17). This poor vascularization contributes to the high risk of impaired healing in osteochondral injuries of the talus, making their treatment particularly challenging (18). The case we presented involved an acute osteochondral fracture. Despite the small size of the osteochondral fragment, it retained attached cancellous bone, which was expected to promote healing following reduction and fixation. This approach helps prevent the risk of further cartilage damage due to local hyaline cartilage loss, chronic residual pain, and the potential progression to ankle arthritis.

This report presents a case of antegrade Kirschner wire fixation for acute small OCFs of the talus, resulting in favorable clinical and radiological outcomes at the final follow-up. The study also provided the details of the specific surgical technique used here.

## Case report

**Figure 1:**
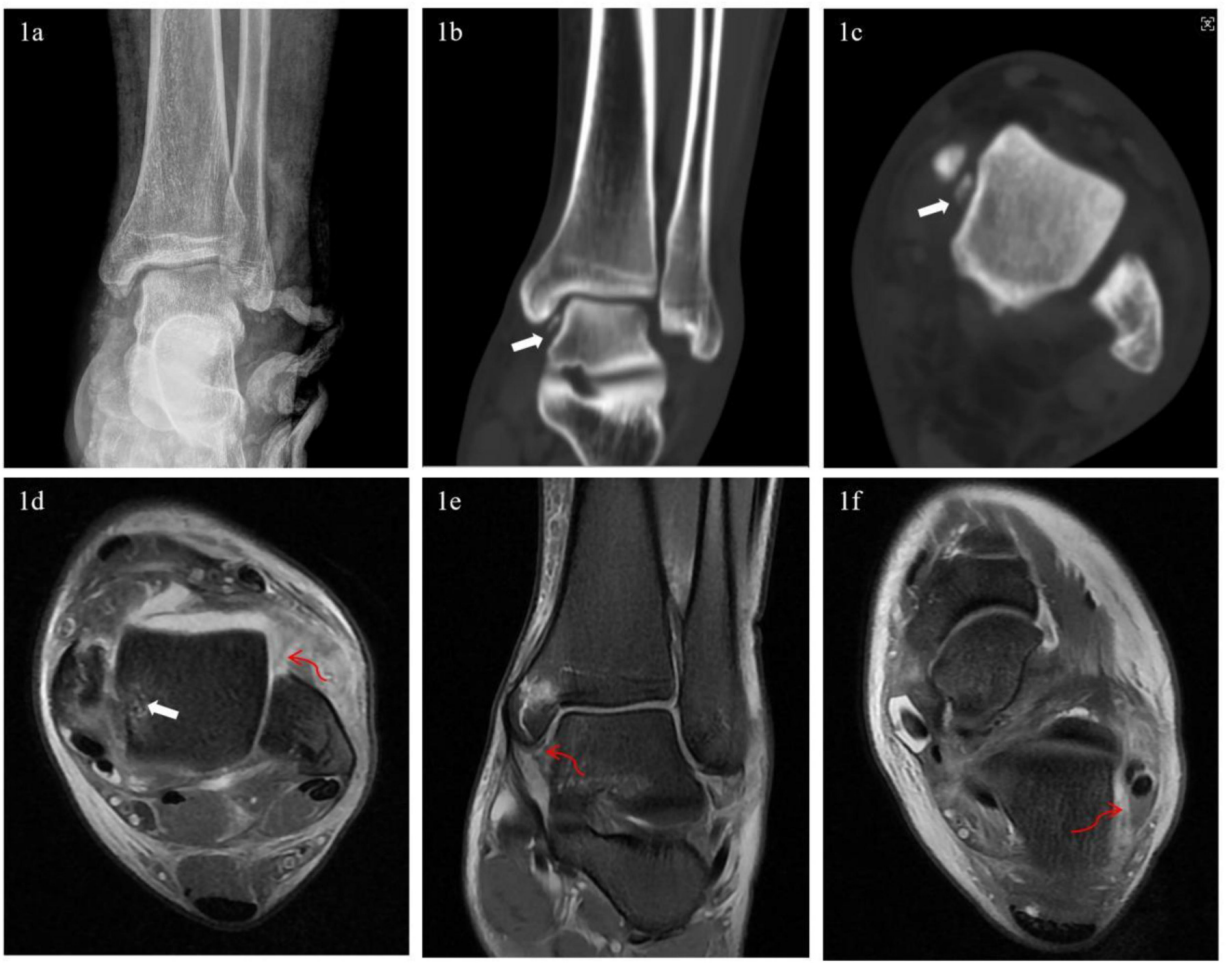
Preoperative imaging of the patient. **(a)** shows an anteroposterior x-ray of the left ankle with a fracture of the medial malleolus. **(b)** and **(c)** present coronal and axial CT images of the left ankle, highlighting a small bone fragment in the medial joint space. **(d)** displays an axial MRI of the left ankle, revealing bone marrow edema in the talus and rupture of the anterior talofibular ligament. **(e)** shows a coronal MRI of the left ankle, demonstrating a tear of the deltoid ligament, while **(f)** provides an axial MRI image depicting the rupture of the calcaneofibular ligament.

A 20-year-old male patient arrived at the emergency department with a two-day history of left ankle sprain sustained during a basketball game. The patient had initially sought treatment at a local facility, where radiographic imaging (anteroposterior and lateral views) of the left ankle revealed a fracture of the medial malleolus (Figure 1a). A cast was applied for immobilization. On physical examination, there was marked swelling of the left ankle joint, accompanied by significant tenderness over both the lateral and medial aspects. The patient was unable to bear weight and required a wheelchair for ambulation. Additional imaging, including magnetic resonance imaging (MRI) and computed tomography (CT), was conducted. The CT scan of the left ankle identified a fracture of the medial malleolus with small bony fragments in the medial joint space (white arrows in Figures 1b, c), suggesting the possibility of a deltoid ligament avulsion fracture. The MRI revealed bone marrow edema in the medial talus and tears in the anterior talofibular ligament, deltoid ligament, and calcaneofibular ligament (red arrows in Figures 1d–f).

**Figure 2:**
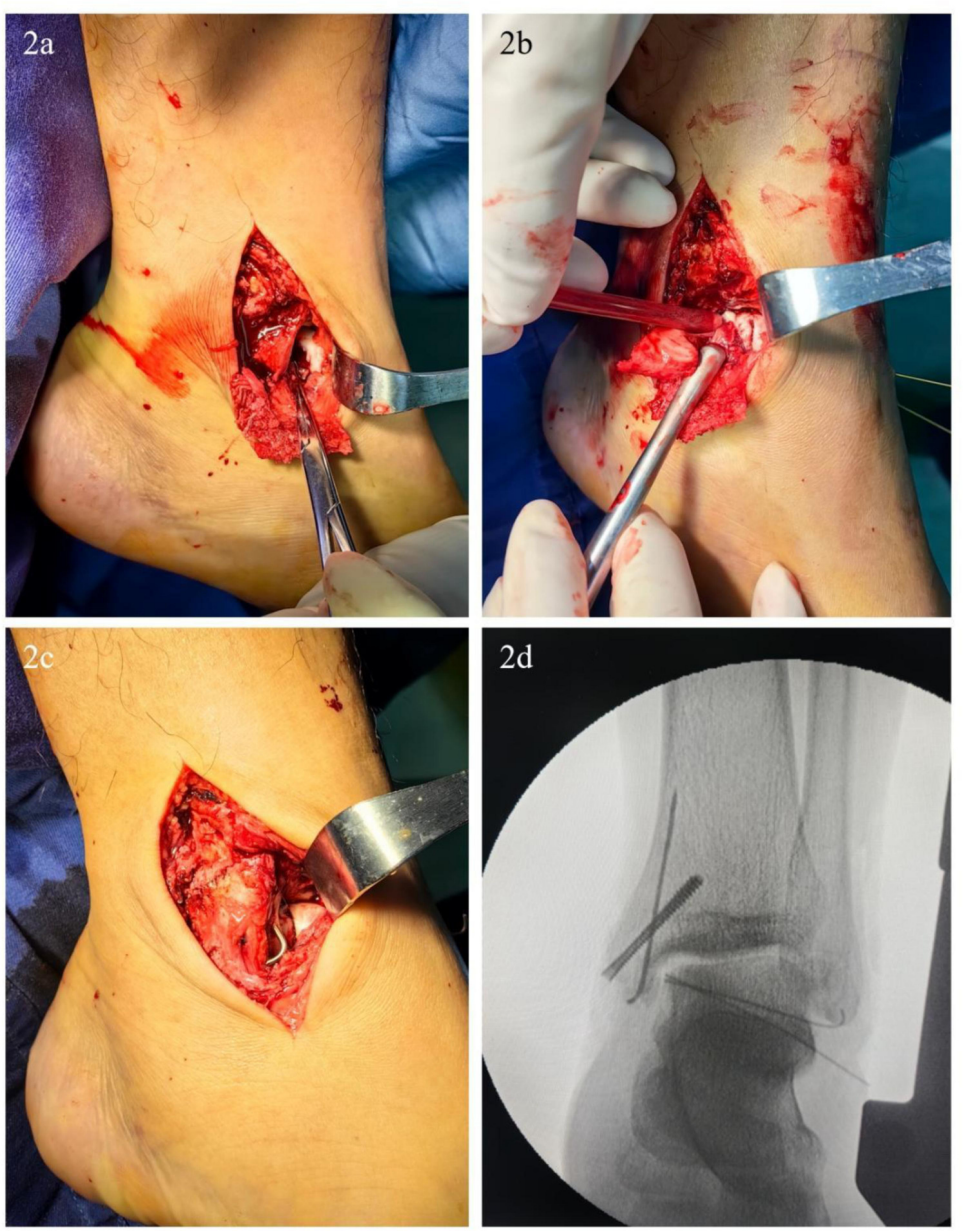
Intraoperative anatomical reduction and fixation of the talar osteochondral fracture fragment. **(a)** shows the intraoperative observation of a talar osteochondral defect located at the shoulder of the talus in Zone 4. **(b)** depicts the reduction and fixation of the talar osteochondral fracture fragment using two 1.0 mm Kirschner wires in an antegrade direction, after exposing the MMF. **(c)** illustrates the reduction and fixation of the MMF. Finally, **(d)** presents intraoperative fluoroscopy confirming satisfactory fracture reduction as well as fixation of the osteochondral fragment and the medial malleolus.

A medial incision was performed at the left ankle to expose the medial malleolus fracture (MMF) and the medial joint space. During the procedure, a tear of the deep deltoid ligament was observed, along with a shoulder-type osteochondral fracture of the talus in the fourth zone (19), as depicted in Figure 2a. The osteochondral fragment was significantly displaced and free, corresponding to a stage IV injury (10). The fractured segment of the medial malleolus was gently retracted to adequately expose the talar osteochondral defect. The talar osteochondral fragment measured approximately 10 × 4 mm. After anatomically reducing the osteochondral fragment, two 1.0-mm Kirschner wires were inserted antegrade and passed through the opposite skin. These wires were then removed retrogradely from the opposite skin, ensuring that the tail of the Kirschner wire was flush with the articular surface (Figure 2b). Confirmation of the osteochondral fragment fixation stability was achieved. Following the reduction of the MMF, two cannulated screws and one 1.5-mm Kirschner wire were used for fixation (Figure 2c). A suture anchor was placed in the intercollicular groove of the medial malleolus, and the deep fibers of the deltoid ligament were repaired. A curved lateral incision was then made on the left ankle, revealing a rupture of the anterior talofibular ligament and calcaneofibular ligament. Two anchors were inserted at the tip of the lateral malleolus for repair of the anterior talofibular and calcaneofibular ligaments to stabilize the ankle joint. Intraoperative fluoroscopy confirmed satisfactory reduction and fixation of the fractures (Figure 2d). After irrigating the wound with 1,000 mL of saline, the incision was closed in layers, and a neutral position cast was applied to the ankle joint.

**Figure 3:**
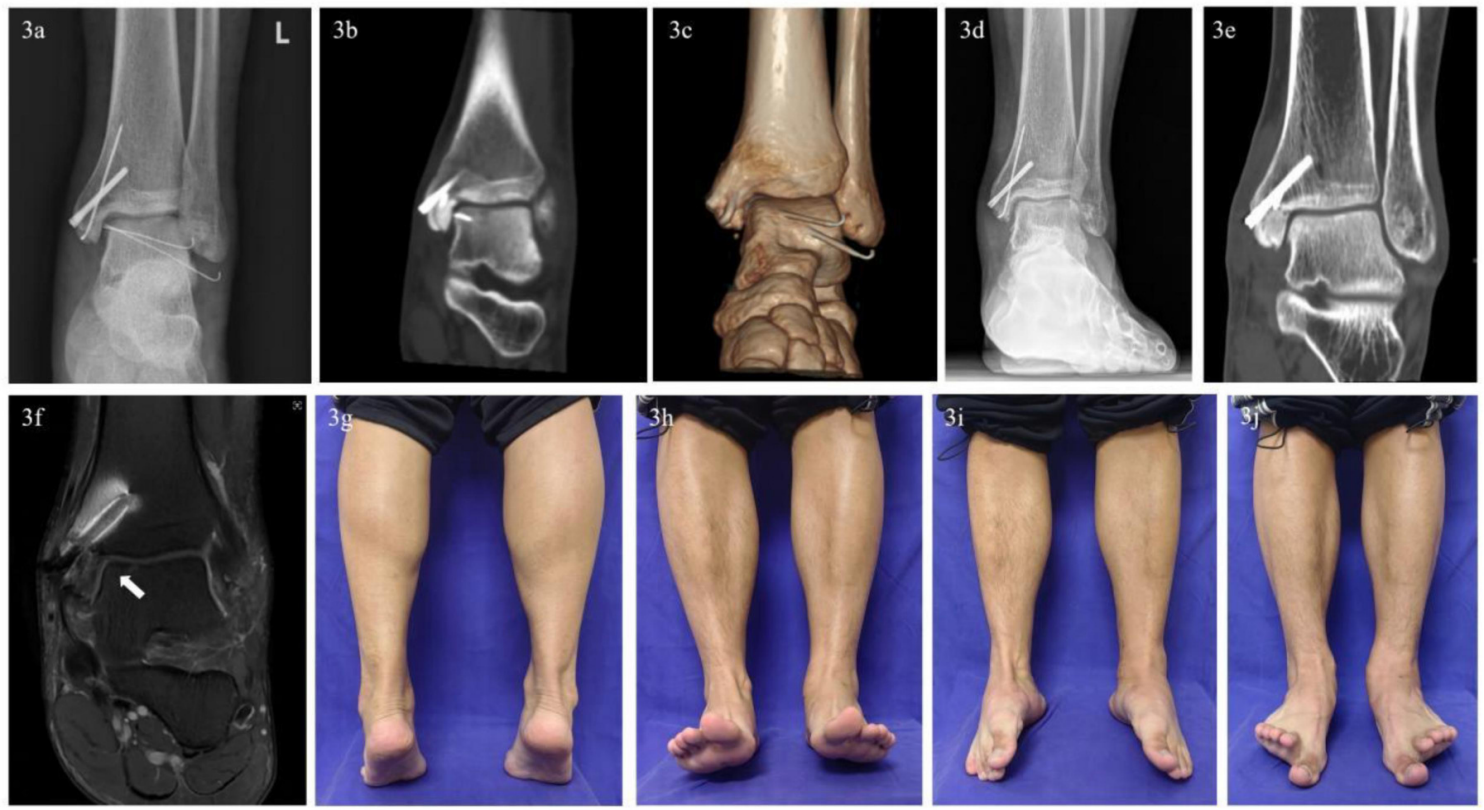
Postoperative follow-up**:** imaging performed two months postoperatively **(a–c)** confirmed satisfactory healing of the osteochondral fragment of the talus. At one year postoperatively, a weight-bearing anteroposterior x-ray **(d)** showed a well-aligned ankle joint. Further CT and MRI scans **(e, f)** revealed proper healing of the talar cartilage, with resolution of bone marrow edema signals in the talus. Functional evaluation at one year **(g–j)** demonstrated excellent recovery of ankle joint function.

Postoperative immobilization of the ankle joint in a neutral position was maintained for two months using a plaster cast. This non-weight-bearing period allows initial healing of both the MMF and the osteochondral fragment without shear stress. During this period, active toe movement and Straight-Leg-Raising movement are allowed. At the two-month follow-up, radiological imaging confirmed the healing of the MMF and the osteochondral fragment of the talus (Figures 3a–c). The Kirschner wires were removed percutaneously*,* and the patient began gradual weight-bearing and start to manual or shock wave release to increase the range of motion of the ankle joint. By three months postoperatively, the patient was able to walk independently, and then some intensive training is allowed, such as jogging, swimming, lower limb strength training. One-year follow-up imaging showed satisfactory restoration of the ankle joint alignment and successful osteochondral healing of the talus (Figures 3d, e). MRI of the left ankle demonstrated resolution of bone marrow edema (Figure 3f). The patient's ankle joint range of motion improved significantly, returning to pre-injury activity levels (Figures 3g–j). The visual analog scale (VAS) score was 0, the American Orthopedic Foot and Ankle Society (AOFAS) score was 100 (20), and the Karlsson-Peterson score during activity was 95 (21).

## Discussion

Flick et al. (1) reported that 98% of lateral talar cartilage injuries are attributed to ankle sprains, typically caused by shear forces when the ankle is dorsiflexed and inverted. However, 70% of medial talar cartilage injuries result from axial loading with the ankle in a plantarflexed, inverted position. In the current case, the injury occurred while the patient was jumping during a basketball game and landed on another player's foot. The ankle sprain occurred with the joint in a plantarflexed and inverted position, and the axial load upon landing resulted in a medial talar cartilage fracture in Zone 4. The injuries included tears to the anterior talofibular ligament, calcaneofibular ligament, and the deep portion of the deltoid ligament, along with an MMF. Based on the Lauge-Hansen classification system (22), the ankle fracture was identified as a supination-adduction type.

Talar OCFs are often underdiagnosed, particularly when involving small osteochondral fragments. The literature reports a preoperative diagnostic rate of only 43% with x-ray imaging (1). While CT scans can detect larger osteochondral fragments, smaller fragments, and isolated talar cartilage injuries remain challenging to identify. In this case, a small bone fragment was observed preoperatively in the medial ankle joint space; however, the talar cartilage defect was not visible due to the fragment's small size. This led to the bone fragment being misinterpreted as an avulsion fracture of the deltoid ligament. The preoperative MRI provided crucial insights, identifying bone marrow edema in the medial dome of the talus on T2-weighted imaging and confirming tears in the anterior talofibular ligament, calcaneofibular ligament, and deltoid ligament. Thus, MRI is essential for diagnosing small talar OCFs. Therefore, preoperative CT showed intra-articular bony fragments, which should raise suspicion of OCF rather than deltoid ligament avulsion, since the latter typically involves the medial malleolar tip rather than the talar dome. Although MRI did not directly visualize the tiny cartilage fragment, the bone marrow edema in the medial talar dome provided an important clue, and the definitive diagnosis was confirmed intraoperatively. Postoperative follow-up is critical for assessing surgical outcomes by monitoring changes in bone marrow edema signals (23). In this case, a 1-year postoperative MRI showed a significant resolution of the bone marrow edema signal on T2-weighted images, reflecting a favorable surgical outcome.

The management of talar OCFs includes both conservative and surgical approaches. Canale and Belding et al. (24) recommended that medial talar osteochondral injuries classified as Berndt and Harty (10) stage I, II, and III can be treated conservatively, whereas stage IV and stage III lateral talar osteochondral injuries typically require surgical intervention. Larger osteochondral fragments typically require internal fixation, commonly achieved using headless screws, absorbable screws, Kirschner wires, or bone pegs (12–14, 25). Nash and Baker et al. (26) reported successful outcomes using two 0.028-inch Kirschner wires to fix stage III lateral talar OCFs. However, they highlighted the risks associated with smooth Kirschner wires, including malposition, displacement, and nonunion, leading them to discontinue this method for later cases. Park and Choi (14) used bone peg treatment for talus fractures with osteochondral fractures in Zones 5 and 6. In their cases, the osteochondral fragments measured 8 × 10 mm and 6 × 7 mm in Case 1 and 7 × 10 mm in Case 2. However, all osteochondral fragments were located on the lateral side of the talus and were not associated with ankle fractures. Furthermore, the fragments could be directly exposed during surgery without requiring osteotomy. Despite their small size, the osteochondral fragments retained intact subchondral bone. In this case, the talar osteochondral fragment measured approximately 10 × 4 mm, with a small size and thin cartilage that rendered other fixation methods unsuitable. During surgery, levering the MMF site created an effect similar to a medial malleolar osteotomy, providing adequate exposure to the talar cartilage defect. The talar cartilage fragment was anatomically reduced, and two 1.0-mm Kirschner wires were inserted for antegrade fixation. Once the Kirschner wires exited the opposite skin, a wire driver/forceps was used to pull the wires retrogradely through the opposite skin, ensuring that the tail ends were flush with the cartilage surface under direct visualization. Unlike previous reports using Kirschner wires for lateral talar OCFs without ankle fracture, our technique addresses a medial OCF in the setting of MMF, leveraging the fracture window for direct visualization. The 10 × 4 mm fragment size and the antegrade fixation with subsequent percutaneous removal represent a refined indication for this approach, though further comparative studies are warranted. Postoperatively, the ankle joint was immobilized in a neutral position for two months. Follow-up imaging at two months confirmed healing of the osteochondral fragment, after which the Kirschner wires were removed percutaneously. Therefore, for patients with small osteochondral fragments on the medial talus accompanied by a medial malleolar fracture, antegrade Kirschner wire fixation is a viable treatment option for selected patients with small medial talar OCFs accompanied by MMF.

The antegrade Kirschner wire fixation technique for acute OCFs of the talus offers several key advantages: (1) It stabilizes small osteochondral fragments containing cancellous bone while preserving the native hyaline cartilage, minimizing the likelihood of post-traumatic osteoarthritis associated with talar cartilage damage. (2) The fixation material is cost-efficient. (3) Although smooth Kirschner wires can be prone to displacement, the antegrade technique provides a long fixation path through the talar body, ensuring stability. (4) The MMF site allows optimal exposure of the talar cartilage defect. This enables accurate reduction of the osteochondral fragment under direct visualization and adjustment of the wire's tail to be flush with the cartilage surface, preventing protrusion and potential damage to the medial malleolar cartilage. (5) Since the opposite end of the Kirschner wire exits through the skin, it can be easily removed percutaneously once imaging confirms osteochondral fragment healing at two months. This approach prevents wire slippage within the talus and reduces the risk of injury to the medial malleolar cartilage.

In summary, this case underscores several critical aspects of treatment: (1) The presence of small bone fragments within the joint space in ankle fractures should raise suspicion of talar OCFs. Comprehensive imaging, including CT and MRI, is crucial to ensure accurate diagnosis. (2) During open reduction and internal fixation of ankle fractures, thorough exposure of both the medial and lateral joint spaces is advised to evaluate the condition of the talar cartilage. (3) When using a Kirschner wire for fixation of a talar cartilage fragment, it is essential to maintain the anatomical reduction of the fragile fragment throughout the procedure with a small vascular clamp. Careful handling is imperative to avoid cartilage fragmentation.

In conclusion, the antegrade Kirschner wire fixation technique has proven to be a successful surgical approach for managing acute small OCFs of the talus. This method effectively preserves hyaline cartilage, promotes the restoration of ankle joint function, reduces the risk of post-traumatic osteoarthritis, and enhances the long-term durability of the ankle joint. Given these advantages, this technique is a valuable option for treating such fractures and holds potential for broader clinical application.

Regarding treatment, we used the antegrade Kirschner wire fixation technique can fix very small acute OCFs, and it can be easily removed percutaneously once imaging confirms the osteochondral fragment is healing, so that no intra-articular hardware remains in the talus. In our patient, good function was maintained at 1 year after surgery. However, there is a risk of Kirschner's breakage and displacement of the OCFs. And, a potential concern is articular cartilage abrasion by the smooth Kirschner wires during the 2-month immobilization period, despite the flush placement. Additionally, pin-tract infection or wire migration remains a risk that requires close follow-up.Therefore, the more case observations and longer-term follow-ups are still needed to verify the safety of this surgical procedure.

## Data Availability

The raw data supporting the conclusions of this article will be made available by the authors, without undue reservation.
